# Super-Hydrophobic Polyurethane/Activated Biochar Composites with Polydimethylsiloxane Coating for High-Efficiency Organic Liquid Uptake

**DOI:** 10.3390/ma19020415

**Published:** 2026-01-21

**Authors:** Rafik Elarslene Dra, Badra Mahida, Malika Medjahdi, Belaid Mechab, Nadia Ramdani, Dominique Baillis

**Affiliations:** 1 Energy and Process Engineering Department, Djillali Liabes University of Sidi Bel Abbes, Sidi Bel Abbes 22000, Algeria; dra.rafik@gmail.com; 2 LRTFM Laboratory, ENPO, Oran 31000, Algeria; badra_mahida@yahoo.fr; 3 APELEC Laboratory, Djillali Liabes University of Sidi Bel Abbes, Sidi Bel Abbes 22000, Algeria; ramdani.nadia73@gmail.com; 4 LMPM Laboratory, Djillali Liabes University of Sidi Bel Abbes, Sidi Bel Abbes 22000, Algeria; bmechab@yahoo.fr; 5 LaMCoS, INSA-Lyon, CNRS UMR5259, 69621 Villeurbanne, France; dominique.baillis@insa-lyon.fr

**Keywords:** marine algae biochar, PDMS coating, hydrophobic sorbent, organic liquid, contaminated water

## Abstract

The aim of this work is to develop structurally enhanced and highly hydrophobic polyurethane (PU) foams for the efficient remediation of liquid organic pollutants. For this purpose, PU foams were modified with renewable activated biochar derived from marine algae (AC) and a hydrophobic polydimethylsiloxane (PDMS) coating, producing four systems: pristine PU, PU-AC, PU/PDMS, and the hybrid PU-AC/PDMS composite. The study evaluates how AC incorporation and PDMS surface functionalization influence the microstructure, chemical composition, wettability, thermal stability, and sorption behavior of the foams. SEM images revealed progressive reductions in pore size from 420 ± 80 μm (PU) to 360 ± 85 μm (PU-AC/PDMS), with AC introducing heterogeneity while PDMS preserved open-cell morphology. FTIR confirmed the presence of urethane linkages, carbonaceous structures, and PDMS siloxane groups. Surface hydrophobicity increased markedly from 88.53° (PU) to 148.25° (PU-AC/PDMS). TGA results showed that PDMS improved thermal stability through silica-rich char formation, whereas AC slightly lowered degradation onset. Sorption tests using petroleum-derived oils and hydrophobic organic liquids demonstrated a consistent performance hierarchy (PU < PU/PDMS < PU-AC < PU-AC/PDMS). The ternary composite achieved the highest uptake capacities, reaching 44–56 g/g for oils and up to 35 g/g for hydrophobic solvents, while maintaining reusability. These findings demonstrate that combining activated biochar with PDMS significantly enhances the functional properties of PU foams, offering an efficient and sustainable material for oil–water separation and organic pollutant remediation.

## 1. Introduction

The persistent problem of hydrophobic organic liquid and petroleum product leaks is a major threat to the environment [1]. The fast spread of hydrocarbons (like gasoline, gasoil, etc.) and hydrophobic organic liquid (like toluene, cyclohexane, etc.) on water surfaces necessitates swift action to forestall their long-term pollution of ecosystems [2]. Porous polymeric materials that can selectively absorb organic liquids while resisting water intrusion have recently attracted a lot of attention from researchers looking for solutions to these kinds of problems [3,4]. The pliability, low density, and linked pore network of polyurethane (PU) sponges set them apart from the others [5,6,7,8]. The chemical stability and hydrophobicity of pure PU are inadequate for effective oil–water separation, however [9,10,11,12,13]. This is why a lot of work has gone into improving PU by adding hydrophobic coatings and carbon-based fillers, which make it more durable, increase adsorption selectivity, and make the surface rougher [14,15,16,17]. The low surface energy, transparency, and chemical resistance of polydimethylsiloxane (PDMS) make it one of the most utilized hydrophobic agents in the literature [18]. Activated carbon [19], graphene [17,20,21], carbon nanotubes (CNTs) [9,22,23], and carbon black are some of the carbon compounds that have been added to PU sponges to enhance their surface roughness and increase their oil affinity. In several of these experiments, carbon particles are anchored to PU substrates using a polydopamine (PDA) adhesive layer, a process known as dopamine-assisted surface functionalization [5]. Some use methods for ultrasonic dispersion and coating to cover the outside of a synthetic PU sponge with carbon nanoparticles [6,24,25,26,27]. The carbon fillers mostly cling to the exterior sponge skeleton, which is a drawback of these post-fabrication coating techniques, even if they are efficient for enhancing wettability and adsorption performance. Consequently, they suffer from reduced durability and compromised long-term reusability due to their potential to detach when subjected to repetitive compression, solvent cleaning, or mechanical stress. When nanocarbons are physically confined within the pores of the PU or very weakly bound, this instability becomes much more apparent. Unlike these earlier approaches, a new and more robust method of modification is presented in this paper. The composite shows better mechanical integrity and reduces the danger of carbon particle loss during reuse by integrating the carbon phase during polymerization instead of depositing it afterwards. An environmentally conscious and sustainable substitute for man-made carbon nanoparticles is biochar, which is generated from algae. Improving the absorption capabilities of PU sponges is a good use of its inherently porous microstructure, high surface activity, and strong affinity for organic liquids. A durable hydrophobic layer is formed by coating the composite sponge with PDMS after in situ biochar absorption. This layer enhances water repellence and guarantees selective uptake of light oils and organic solvents. Incorporating biochar into the PU matrix and treating the surface with PDMS together create an absorbent material that is both structurally stable and very efficient. Biochar, a component of PUs made from marine algae, provides improved mechanical anchoring and long-term stability as compared to traditional post-coated systems. The PDMS layer creates a strong and long-lasting hydrophobic surface, while the enhanced porosity structure improves the sponge’s affinity for organic liquids. This causes the material to absorb liquids including ethanol, acetone, gasoline, gasoil, and Algerian crude oil quickly and selectively. Adding renewable algae charcoal to the mix makes it even more eco-friendly. All these characteristics show that there is an easy way to make absorbent sponges that can be used to separate and recover organic solvents and light oils efficiently. Therefore, the objective of this work is to develop a structurally stable and highly efficient polyurethane-based absorbent for oil and organic solvent remediation by combining in situ incorporation of algae-derived biochar within the PU matrix and post-surface modification with PDMS. Specifically, this study aims to (i) improve the mechanical anchoring and durability of carbon fillers by integrating biochar during PU polymerization, (ii) enhance surface hydrophobicity and oil selectivity through PDMS coating, and (iii) evaluate the adsorption capacity, reusability, and stability of the resulting composite toward various organic liquids and crude oil.

## 2. Materials and Methods

### 2.1. Materials

Polyol (HO-(R-O)n-H) and isocyanate (C_15_H_10_N_2_O_2_) from used for polyurethane synthesis were supplied by the “Style Mousse” mattress production company, Es-Senia, Oran, Algeria originally from Ludwigshafen, Allemande. Marine algae biomass, employed as the precursor for activated biochar, was collected from Stadia Beach in Mostaganem, Algeria. Polydimethylsiloxane (PDMS, Sylgard 184; commercial grade) and its curing agent, as well as hexane, toluene, and paraffin oil (analytical grade, purity ≥ 99%), were purchased from Sigma-Aldrich (Burlington, MA, USA). All chemicals were used without further purification. The oils employed in the sorption experiments were provided by the Arzew refinery (Oran, Algeria). The crude oil used had a kinematic viscosity of 3.61 cSt and a density of 0.80 g/cm^3^ at 20 °C.

### 2.2. Methods

#### Activated Biochar

To improve the sorption capacity of the foam, activated biochar derived from marine algae was integrated into the PU foam composition. The algae, collected from Stadia Beach in Mostaganem, Algeria, underwent both thermal (at 600 °C for 2 h) and chemical activation (using H_3_PO_4_ from Sigma-Aldrich (USA): Phosphoric acid activation promotes dehydration, crosslinking, and bond-cleavage reactions during pyrolysis, leading to the development of a porous carbon structure enriched with oxygen- and phosphorus-containing functional groups such as –OH, –COOH, and P–O–C. These groups increase surface polarity) [28]. The resulting biochar properties is shown in Table 1.

These values highlight the high specific surface area, significant porosity, and excellent adsorption potential of the algae-derived activated carbon. The iodine and methylene blue indices further confirm its capacity to adsorb both micropollutants and larger organic molecules. The chemical functionalities present on the biochar surface were confirmed through FTIR spectroscopy, while the morphology was characterized using SEM (in Supplementary Data). These analyses revealed the presence of hydroxyl, carboxyl, and carbonyl groups, which contribute to enhanced adsorption capacity, along with a porous surface morphology suitable for composite integration.

### 2.3. Synthesis of PU-AC Composite Foam (In Situ Incorporation)

**Figure 1:**
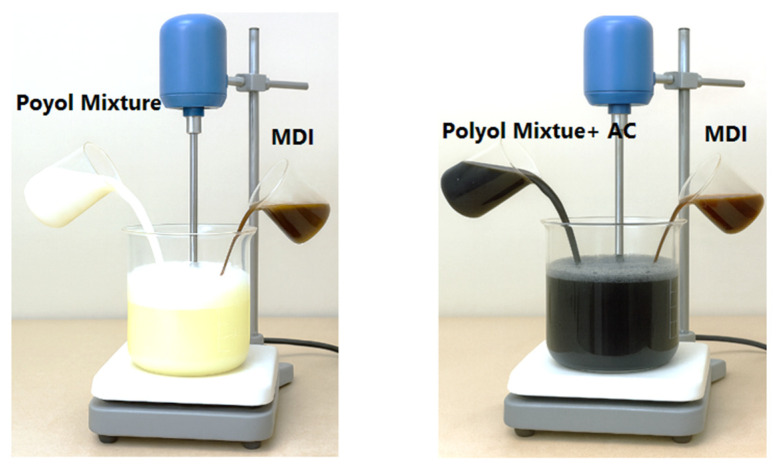
Procedure for PU and PU-AC Sample Preparation.

Polyurethane foams were prepared using a one-shot process based on the reaction of methylene diphenyl diisocyanate (MDI) with a polyol mixture containing water (chemical blowing agent), silicone glycol (surfactant), n-pentane (physical blowing agent), and N,N-dimethylcyclohexylamine (catalyst), following the formulation in Table 2. The isocyanate and polyol components were mixed at an NCO/OH ratio of 1.1 to obtain the reference PU foam [29] (Figure 1).

For the preparation of composite samples, algae-derived activated biochar was incorporated directly into the polyol phase before foaming. A fixed mass of biochar (2.0 g, particle size 200 μm) was dispersed into 10 g of polyol under mechanical stirring and then ultrasonically homogenized. After complete dispersion, the required amount of MDI was added, and the mixture was stirred for 10–20 s to initiate the foaming reaction.

The reactive blend was immediately poured into open molds and allowed to expand and cure at room temperature for 48 h. The foams were then demolded, rinsed with deionized water, and dried. The resulting composite materials, containing biochar uniformly embedded within the PU matrix, were designated PU-AC.

### 2.4. Preparation of PDMS-Coated Foams (PU–PDMS and PU-AC–PDMS)

**Figure 2:**
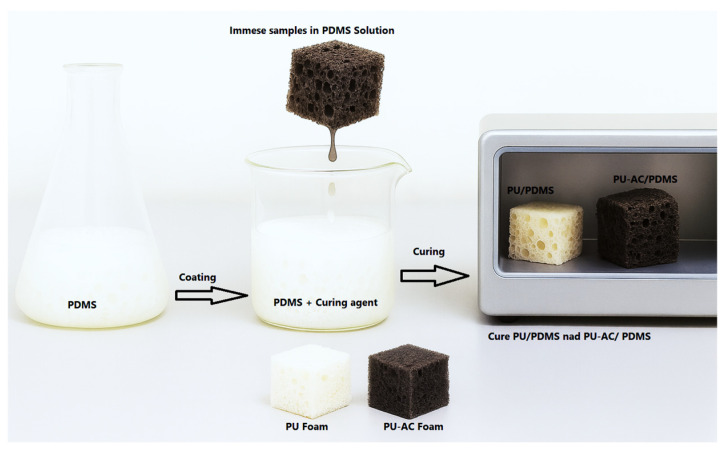
Procedure for PDMS-coated foams.

Polyurethane (PU) and polyurethane-activated biochar (PU-AC) foams were cut into homogeneous 2 × 2 × 2 cm cubes using a clean, sharp stainless-steel blade to achieve consistent geometry for coating and sorption testing. Surface hydrophobization was achieved by mixing polydimethylsiloxane (PDMS) prepolymer and curing agent (Sylgard 184) in a 10:1 mass ratio and stirring until homogenous. The mixture was then diluted in hexane to provide a low-viscosity coating solution (10 wt% PDMS). Each foam cube was completely submerged in the PDMS solution for 10 min. While immersed, the samples were gently pressed several times to eliminate trapped air and enable the PDMS solution to permeate the open-cell network. After impregnation, the cubes were removed and allowed to drain at room temperature for several minutes to remove any excess PDMS. The coated foams were then cured in an oven at 80 °C for 2 h to ensure that the silicone layer was completely crosslinked (Figure 2). After curing, the samples were cooled to room temperature and kept in sealed containers until later use [17,18]. The resultant materials were termed as PU/PDMS and PU-AC/PDMS.

### 2.5. Structural and Physicochemical Characterization

#### 2.5.1. SEM Analysis

The microstructure, pore morphology, and distribution of activated biochar within the polyurethane matrix were examined using scanning electron microscopy (SEM) (instrument model Nova NanoSEM 450 (FEI, Hillsboro, OR, USA)) [15,30,31,32,33,34]. Foam samples were cut into small sections and dried before imaging.

#### 2.5.2. FTIR and X-Ray Photoelectron Spectroscopy

Fourier Transform Infrared (CFTIR) spectra were recorded using a spectrometer (model Nicolet iS10 FTIR spectrometer; Thermo Fisher Scientific, Madison, WI, USA) in the range of 4000–400 cm^−1^ with a resolution of 4 cm^−1^. All samples were analyzed in ATR mode without additional preparation [15,34].

X-ray photoelectron Spectroscopy (XPS) measurements were performed using a spectrometer with a monochromatic Al Kα radiation source (1486.6 eV) under ultra-high vacuum conditions. Wide-scan survey spectra were recorded to determine the elemental composition of the sample surfaces. Binding energies were referenced to the C 1s peak at 284.8 eV for charge correction. All spectral analyses were conducted using CasaXPS software (version 2.3.25, Casa Software Ltd., Teignmouth, UK).

#### 2.5.3. Wettability Measurements

Static water contact angles (WCA) were measured using a Krüss DSA25E Drop Shape Analyzer (KRÜSS GmbH, Hamburg, Germany). The contact angle measurements were performed on representative surface regions where the foam structure was sufficiently uniform, in order to minimize the direct influence of macroscopic pores. During the experiments, the samples were placed inside a glass measurement chamber to minimize environmental disturbances. A 5-μL droplet of deionized water was deposited on the foam surface using a calibrated microsyringe, and the contact angle was recorded within 2 s to limit liquid penetration into the porous structure [21]. The captured images were analyzed using ImageJ v1.42 software, and the reported WCA values represent the average of three measurements taken at different positions on each sample.

#### 2.5.4. Thermogravimetric Analysis (TGA)

Thermal stability was assessed using thermogravimetric analysis (TGA) performed on a TGA instrument (model TGA 2050 Thermogravimetric Analyzer (TA Instruments, New Castle, DE, USA). Approximately 5–10 mg of each sample was heated from room temperature to 600–800 °C at a rate of 10 °C/min under nitrogen flow [35,36]. The characteristic weight-loss stages associated with PU degradation such as moisture release, hard-segment decomposition, and soft-segment degradation were identified.

### 2.6. Sorption Capacity Tests

#### Sorption Measurements

Foam samples (mass m_0_) were immersed for 60 min in each test liquid: Algerian crude oil, gasoline, gasoil, motor oil, toluene, cyclohexane and paraffin oil. After immersion, samples were removed, drained for 30 s, and weighed (m_sorb_). Sorption capacity (Q) was calculated using (ASTM F726-99 [37]) [19]:

(1) $$Q_{\left(g/g\right)}=\frac{m_{sorb}-m_{0}}{m_{0}}$$

## 3. Results and Discussion

### 3.1. SEM Micrographs

**Figure 3:**
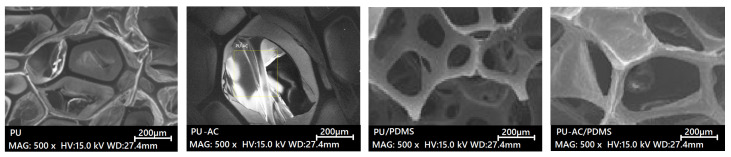
SEM images of PU, PU-AC, PU/PDMS and PU-AC/PDMS.

SEM images (Figure 3) were analyzed to evaluate cell size, pore uniformity, and the degree of activated carbon (AC) integration within the polyurethane (PU) matrix. Comparisons were carried out between unmodified PU, AC-loaded PU (PU-AC), and PDMS-coated samples (PU/PDMS and PU-AC/PDMS) to assess the microstructural changes induced by filler incorporation and surface treatment.

**Figure 4:**
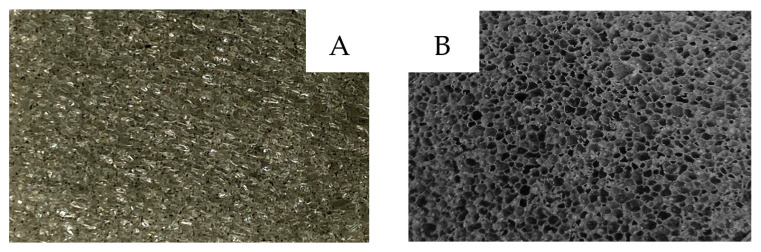
Surface view of Real images of PU-AC (**A**) and PU-AC/PDMS (**B**).

SEM imaging was used to assess pore size, uniformity, and the degree of AC integration within the PU matrix. Pristine PU showed a uniform open-cell morphology with an average pore size of 420 ± 80 μm. Incorporation of activated carbon (PU-AC; Figure 4A) disrupted this structure, leading to less uniform pores, visible AC agglomerates within the cells, and a reduced average pore size of 380 ± 90 μm, indicating increased heterogeneity [14,18,21,38]. This reduction in pore size is attributed to the presence of AC particles acting as heterogeneous nucleation sites during the foaming process and physically restricting bubble growth and coalescence.

In contrast, PDMS-coated PU (PU/PDMS; Figure 4B) maintained a morphology similar to unmodified PU, with pores averaging 410 ± 70 μm, confirming that the coating formed a thin surface layer without obstructing the open-cell network. The dual-modified PU-AC/PDMS sample showed the smallest pore size (360 ± 85 μm) due to combined effects of AC occupation and PDMS encapsulation. Overall, AC primarily altered internal pore geometry, whereas PDMS coating preserved pore openness while stabilizing the filler within the structure.

**Figure 5:**
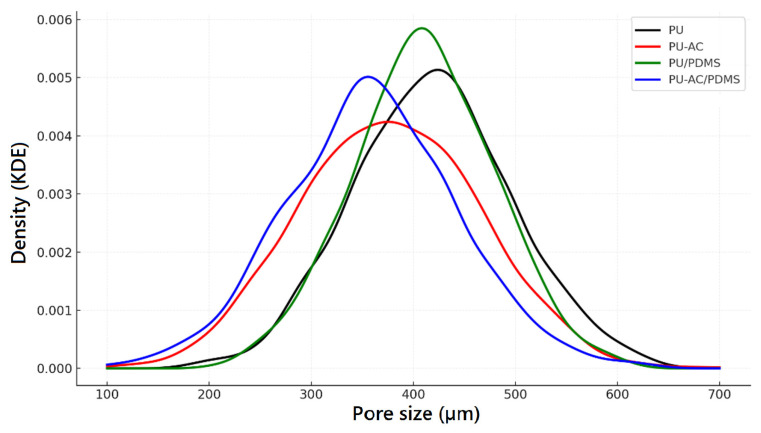
KDE curves of the pore size distributions for PU-based samples.

The Kernel density estimation KDE curves (Figure 5) show that PU has the largest and most uniform pore size distribution, while PU-AC and PU-AC/PDMS exhibit progressively smaller and broader distributions due to filler incorporation. PDMS coating maintains a distribution close to pristine PU, confirming minimal structural disruption.

### 3.2. FTIR and XPS Analysis

FTIR and XPS analyses were combined to investigate the chemical structure, surface composition, and interfacial interactions in pristine PU, PU-AC, PU/PDMS, and PU-AC/PDMS samples.

**Figure 6:**
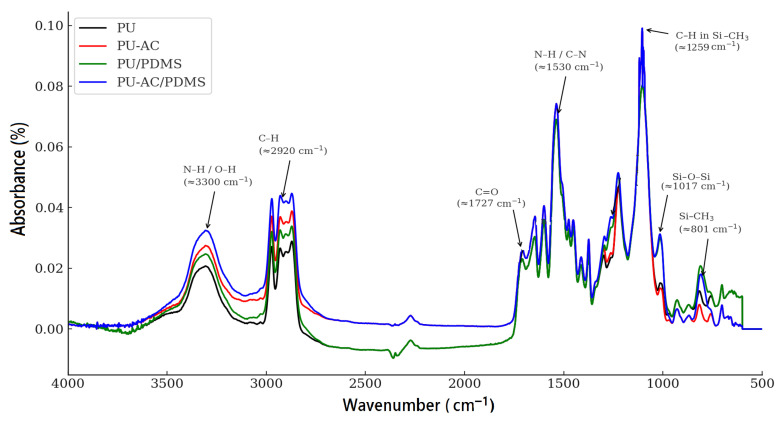
FTIR spectra of PU-based samples.

FTIR spectra (Figure 6) confirms the successful formation of the polyurethane structure for all formulations.

The reference PU spectrum displays the characteristic urethane vibrations, including the broad N–H stretching band centered around ~3300 cm^−1^ [39], aliphatic C–H stretching near 2920 cm^−1^ [40], the strong urethane carbonyl band at ~1727 cm^−1^, and the N–H bending/C–N stretching region near 1530 cm^−1^ [27]. The absorption features between 1100–1200 cm^−1^ correspond to C–O–C stretching, further validating the formation of urethane linkages. Upon addition of activated carbon (PU-AC), notable changes arise in the 1600–1500 cm^−1^ region, where enhanced C=C and aromatic C–H vibrations indicate the presence of carbonaceous structures. Slight increases in the intensity of the carbonyl and N–H related bands also suggest interactions between AC surface functionalities and the PU matrix, likely through hydrogen bonding or restricted chain mobility. Coated samples (PU/PDMS and PU-AC/PDMS) [14] exhibit additional absorption peaks corresponding to polydimethylsiloxane. The appearance of the Si–O–Si asymmetric stretching band in the 1000–1100 cm^−1^ range, along with Si–CH_3_ deformation and rocking vibrations at ~1259 and ~801 cm^−1^, respectively, provides clear evidence of successful PDMS deposition on the PU surfaces. The weak absorption band at ~801 cm^−1^ observed in pristine PU originates from the silicone glycol stabilizer used during synthesis. These bands are more intense in the PU-AC/PDMS sample, suggesting improved PDMS anchoring or coating uniformity in the presence of activated carbon. This enhancement is attributed to the increased surface roughness and higher interfacial compatibility introduced by AC, which likely facilitates stronger interactions between PDMS chains and the modified PU surface.

**Figure 7:**
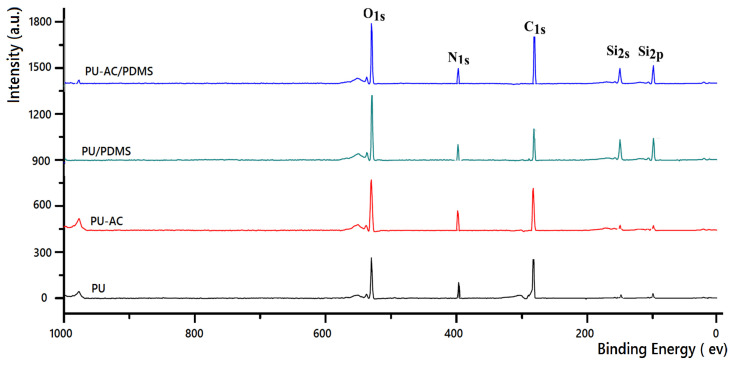
XPS survey spectra of PU-based samples.

X-ray photoelectron spectroscopy (XPS) was used to examine the surface chemical composition of the PU-based samples (Figure 7). All materials show characteristic C 1s, O 1s, and N 1s signals corresponding to the polyurethane backbone. A weak silicon signal observed in pristine PU and PU-AC is attributed to the silicone glycol surfactant used during foam synthesis [41,42]. In contrast, PU/PDMS and PU-AC/PDMS exhibit a markedly higher Si 2s and Si 2p intensity, confirming the formation of a siloxane-rich surface layer. The absence of new chemical states or significant shifts in the N 1s region indicates that activated biochar is physically integrated rather than covalently bonded to the PU matrix. Overall, PDMS modification effectively alters the surface chemistry while preserving the polyurethane structure, which is consistent with the enhanced hydrophobicity and sorption performance of the PU-AC/PDMS composite.

### 3.3. Contact Angle

Unmodified PU exhibited moderate hydrophilicity, while PU-AC samples showed slightly reduced wettability due to the presence of carbon particles. A significant increase in WCA was observed for PU–PDMS and PU-AC–PDMS samples, confirming the formation of a stable, low-surface-energy hydrophobic coating.

**Figure 8:**
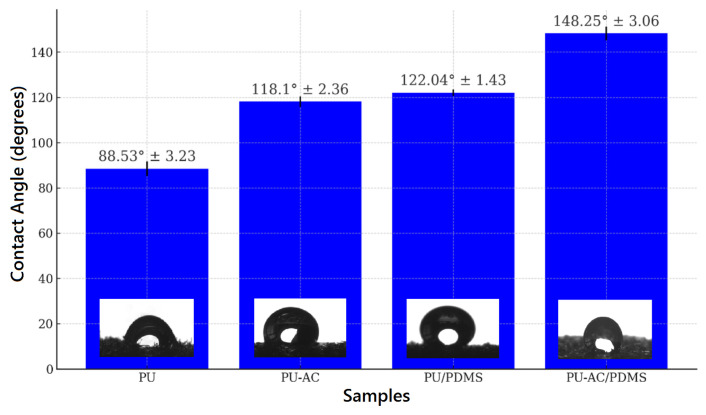
Contact angle variation with error bars.

The contact angle measurements (Figure 8) reveal a progressive increase in hydrophobicity across the modified polyurethane samples. The unmodified PU exhibits a contact angle of 88.53°, indicating moderate hydrophobic behavior. Upon incorporation of activated biochar (PU-AC), the contact angle increases significantly to 118.10°, suggesting that the biochar introduces surface roughness and lowers surface energy, thereby enhancing water repellency. Coating the PU with PDMS (PU/PDMS) further increases the contact angle to 122.04°, consistent with the intrinsically hydrophobic nature of silicone-based materials. The highest contact angle, 148.25°, is observed for PU-AC/PDMS. This value falls in the superhydrophobic regime and indicates a strong synergistic effect between activated biochar and PDMS: the biochar contributes micro/nanotexture, while PDMS provides a low-energy chemical composition. Overall, the contact angle evolution (88.53° → 148.25°) demonstrates that both activated biochar and PDMS play complementary roles in significantly improving the surface hydrophobicity of the polyurethane matrix.

### 3.4. Thermogravimetric Analysis

**Figure 9:**
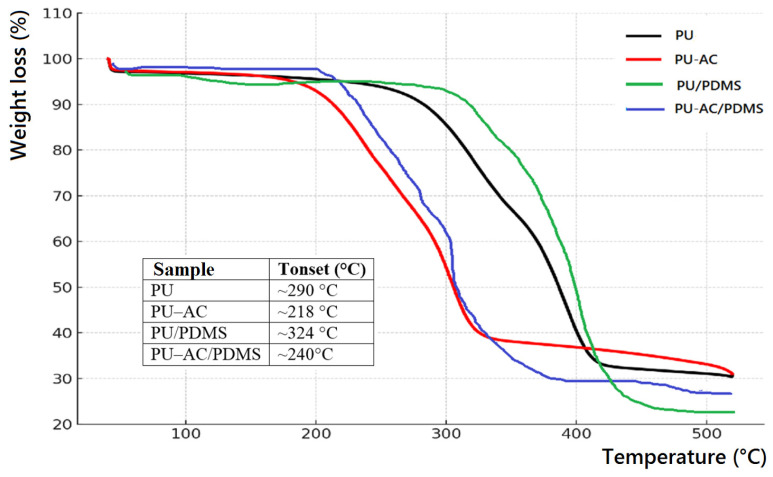
TGA of PU-based samples.

The incorporation of activated biochar was evaluated (Figure 9) by changes in onset degradation temperature and residual mass at high temperature. PDMS-coated samples exhibited increased char residue and slight shifts in thermal-degradation behavior due to the presence of thermally stable silicone components.

The thermal stability of the prepared polyurethane systems was evaluated by thermogravimetric analysis (TGA), and the corresponding weight-loss curves are presented in Figure 9. Pristine PU exhibited a typical two-step degradation profile, with the onset of decomposition (Tonset) defined at 10% mass loss (T_10%_) occurring near 290 °C and a major mass-loss event between 320 and 420 °C. Incorporation of activated carbon (PU-AC) shifted the degradation to slightly lower temperatures (≈218 °C), indicating that AC promotes heat transfer and may catalyze the scission of urethane linkages, resulting in reduced thermal stability. In contrast, the introduction of PDMS into the PU matrix (PU/PDMS) markedly improved the thermal resistance, as evidenced by a delayed onset (≈324 °C) of degradation and a broader decomposition interval. This enhancement is attributed to the high thermal stability of the Si–O–Si backbone and the formation of a silica-rich protective char at elevated temperatures. The hybrid PU-AC/PDMS sample displayed an intermediate behavior with Tonset ≈ 240 °C, reflecting the competing effects of AC-induced catalytic degradation and PDMS-induced stabilization. Overall, the results demonstrate that PDMS contributes significantly to improving the thermal robustness of PU-based networks, while activated carbon tends to facilitate earlier thermal decomposition.

**Figure 10:**
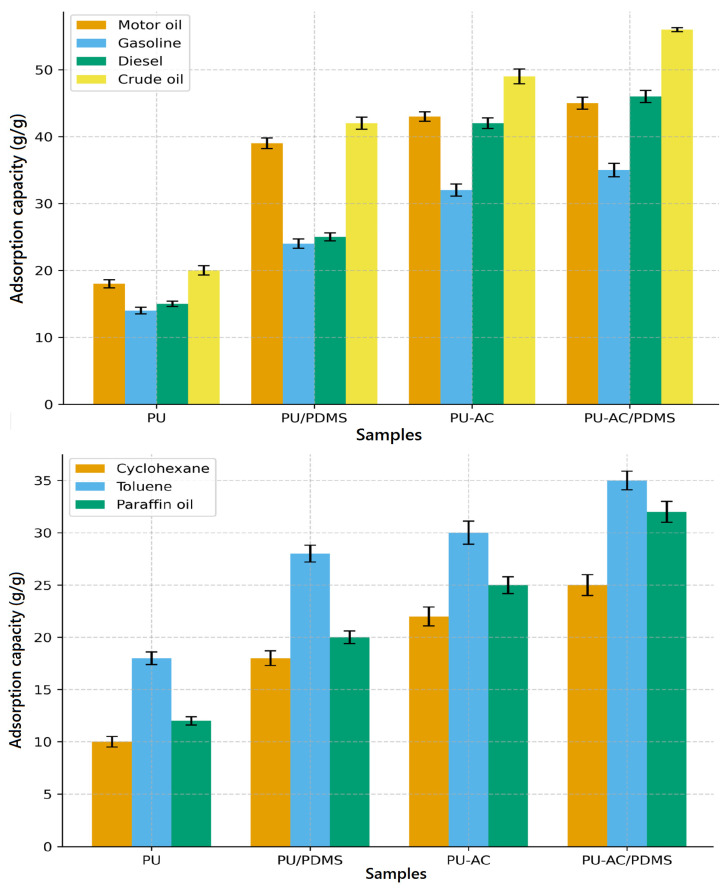
Oils (**first**) and hydrophobic organic liquids (**second**) sorption capacities of PU-based samples.

Chemical modification significantly enhances the sorption performance of polyurethane foams (Figure 10). Neat PU shows the lowest uptake (14–20 g/g for oils) due to its limited hydrophobicity, while PDMS incorporation increases capacities up to ~40 g/g by improving surface hydrophobicity and wetting. Activated carbon further boosts adsorption through increased porosity and surface roughness, enabling deeper infiltration of organic liquids. The PU-AC/PDMS hybrid shows the highest performance, reaching 44–56 g/g for oils and up to 35 g/g for hydrophobic solvents, confirming a strong synergistic effect between PDMS and activated carbon. The overall trend (PU < PU/PDMS < PU-AC < PU-AC/PDMS) highlights the importance of both surface chemistry and hierarchical pore structure in governing adsorption efficiency. Table 3 provides a comparative overview of PU-based composite sorbents previously reported in the literature and those developed in the present work. For comparison, commercial nonwoven polypropylene (PP) sorbents evaluated using ASTM F726-type procedures typically show sorption capacities of 5.3 g/g for diesel, 12.3 g/g for petroleum, and 18.7 g/g for lubricant oil [43]. In contrast, the PU-AC/PDMS composite developed in this study exhibits markedly higher uptake values (Figure 10), reaching approximately 45.8 g/g for diesel, 56.1 g/g for crude oil, and 44.3 g/g for motor oil, corresponding to an improvement of roughly 2.5–8 times over commercial PP sorbents. These results highlight the superior absorption efficiency of the PU-AC/PDMS material for oil spill remediation.

**Figure 11:**
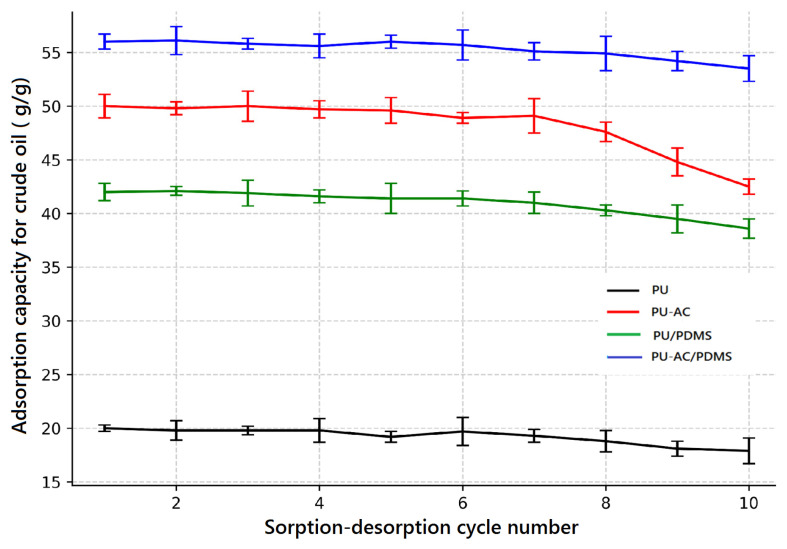
Crude oil cyclic sorption–desorption testing.

The sorption–desorption cycling performance was evaluated using crude oil as a representative pollutant, as shown in Figure 11.

The sorption–desorption cycling performance was evaluated using crude oil as a representative pollutant (Figure 11). Pristine PU shows the lowest adsorption capacity, decreasing slightly from about 20 g/g in the first cycle to 18 g/g after ten cycles, reflecting limited sorption efficiency. The PU/PDMS composite exhibits improved capacity and stability, with crude oil uptake decreasing from approximately 42 g/g to 39 g/g over ten cycles, due to enhanced surface hydrophobicity and elastic recovery.

The PU-AC sample displays a higher initial adsorption capacity of around 50 g/g, but a more noticeable decline occurs after about the sixth cycle, reaching approximately 43 g/g after ten cycles, which can be attributed to filler-induced mechanical fatigue. In contrast, the PU-AC/PDMS composite demonstrates the highest adsorption capacity and cycling stability, with values decreasing only slightly from about 56 g/g to 53 g/g, corresponding to a retention of more than 94%. This high retention confirms that the PDMS coating enhances mechanical robustness during cyclic operation by stabilizing the foam structure and mitigating fatigue effects associated with activated carbon incorporation [45]. These results confirm the synergistic effect of activated carbon incorporation and PDMS coating in enhancing both adsorption performance and reusability for crude oil remediation.

From an environmental perspective, the PU–biochar/PDMS composite shows promising sustainability potential within a life cycle assessment framework. The use of algae-derived biochar reduces reliance on energy-intensive synthetic nanomaterials, while its in situ incorporation enhances durability and reusability, extending service life. Although PDMS is synthetic, its application as a thin coating minimizes material use while significantly improving performance, enabling recovery and potential regeneration and reducing environmental impact compared to single-use sorbents.

## 4. Conclusions

This work demonstrates that the combined in situ incorporation of algae-derived activated biochar and post-surface modification with PDMS is an effective strategy for overcoming the limitations of conventional polyurethane (PU) sorbents. Embedding the biochar during PU polymerization ensures stable filler anchoring and long-term mechanical integrity, leading to controlled pore refinement while preserving the open-cell structure required for rapid mass transfer. The average pore size decreases from 420 ± 80 μm in pristine PU to approximately 360 ± 85 μm in PU-AC/PDMS. Surface modification with PDMS significantly enhances hydrophobicity, increasing the water contact angle from 88.53° for PU to a superhydrophobic value of 148.25° for PU-AC/PDMS, and stabilizes the Cassie–Baxter wetting state through a hierarchical micro–nano architecture. PDMS also improves thermal performance by delaying the onset of degradation from ~290 °C for PU to ~324 °C for PU-AC/PDMS, while the hybrid PU-AC/PDMS exhibits intermediate but improved thermal stability of approximately 240 °C and higher char yields at elevated temperatures. These synergistic structural and chemical effects translate directly into enhanced sorption performance. Whereas pristine PU shows limited oil uptake of 14–20 g/g, PU-AC/PDMS achieves sorption capacities of 44.3 g/g for motor oil, 45.8 g/g for diesel and up to 56.1 g/g for crude oil, as well as uptake values of around 35 g/g for hydrophobic organic solvents. Importantly, these performance levels are maintained over repeated absorption–squeezing cycles, confirming the composite’s mechanical robustness and reusability. Beyond performance improvements, the use of marine algae-derived biochar adds a sustainability dimension by utilizing renewable biomass and reducing dependence on energy-intensive synthetic nanocarbons. From a life-cycle perspective, the enhanced durability, regeneration capability and high sorption efficiency of the PU-AC/PDMS composite suggest a lower environmental footprint compared with single-use or weakly coated sorbents. Overall, this study presents a high-performance, reusable absorbent material and a scalable, environmentally conscious design framework for next-generation polyurethane-based sorbents, with future work focusing on quantitative life-cycle assessment and evaluation under realistic spill conditions.

## Figures and Tables

**Table 1:** Algae activated biochar proprieties.

N°	Proprieties	Values
1	Moisture content (%)	1.972
2	Ash content (%)	1.137
3	Bulk density (g/cm^3^)	0.523
4	Pore volume (cm^3^/g)	0.328
5	Iodine index (mg/g)	945.26
6	methylene blue index (mg/g)	609.794
7	BET surface area (m^2^/g)	524.182
8	pH_PZC_	8.2

**Table 2:** Chemical formulation of base polyurethane foam.

N°	Reagents	Quantity (g)
1	Isocyanate (MDI 4,4-polyphenylene)	10
2	Polyol (polyether polyols)	9
3	Catalyst (N,N-dimethylcyclohexylamine)	0.2
4	Stabilizer (Silicone glycol)	0.4
5	Blowing agent (n-pentane)	0.65
6	Water	1–2

**Table 3:** Comparison between the PU-based materials and tested in this study.

Composite	Crude Oil Sorption Capacity (g/g)	Reference
PU-CNT	41.8 ± 1.0	[9]
PU-AC	38.5 ± 0.2	[39]
PU-graphite	21.5 ± 0.8	[40]
PU-nanoclay	19.3 ± 0.3	[38]
PU-PDMS	48.2 ± 0.6	[14]
PU-Palm fiber	28.9 ± 1.0	[44]
PU-Biochar	42 ± 0.8	[29]
PU-Biochar/PDMS	56.1 ± 0.3	This study

## Data Availability

The original contributions presented in the study are included in the article/Supplementary Material. Further inquiries can be directed to the corresponding author.

## Supplementary Materials

The following supporting information can be downloaded at: https://www.mdpi.com/article/10.3390/ma19020415/s1. Figure S1: FTIR spectrum and SEM image of the used algae activated carbon (ACA); Figure S2: Particle size distributions of algae biochar (differential and cumulative curves); Table S1: Algae-based biochar content elements (* includes mineral residues such as K, Ca, and Mg); Figure S3: Thermogravimetric analysis curves of raw algae and algae-derived activated carbon; Figure S4: High-resolution XPS C 1s spectra of PU-based composites; Figure S5: XPS O 1s spectra; Figure S6: XPS N 1s spectra; Figure S7: XPS Si 2p spectra.
