# Supplementary material for: Super-Hydrophobic Polyurethane/Activated Biochar Composites with Polydimethylsiloxane Coating for High-Efficiency Organic Liquid Uptake
Source: Materials (Basel). 2026 Jan 21;19(2):415. doi: 10.3390/ma19020415 (PMC12843218; doi:10.3390/ma19020415)
Supplement: Supplementary file 1 [file materials-19-00415-s001.zip › materials-4059786-supplementary.pdf]

materials

MDPI

Supplementary Materials

Super-Hydrophobic Polyurethane/Activated Biochar Composites with Polydimethylsiloxane Coating for High-Efficiency Organic Liquid Uptake

Rafik Elarslene Dra [1], Badra Mahida [2], Malika Medjahdi [3,*], Belaid Mechab [4], Nadia Ramdani [3] and Dominique Baillis [5]

[1] Energy and Process Engineering Department, Djillali Liabes University of Sidi Bel Abbes, Sidi Bel Abbes 22000, Algeria
[2] LRTFM Laboratory, ENPO, Oran 31000, Algeria
[3] APELEC Laboratory, Djillali Liabes University of Sidi Bel Abbes, Sidi Bel Abbes 22000, Algeria
[4] LMPM Laboratory, Djillali Liabes University of Sidi Bel Abbes, Sidi Bel Abbes 22000, Algeria
[5] LaMCoS, INSA-Lyon, CNRS UMR5259, 69621 Villeurbanne, France; dominique.baillis@insa-lyon.fr
* Correspondence: mmedjahdi@yahoo.fr

Supplementary Data

The chemical functionalities present on the biochar surface were confirmed through FTIR spectroscopy, as shown in Figure 1.

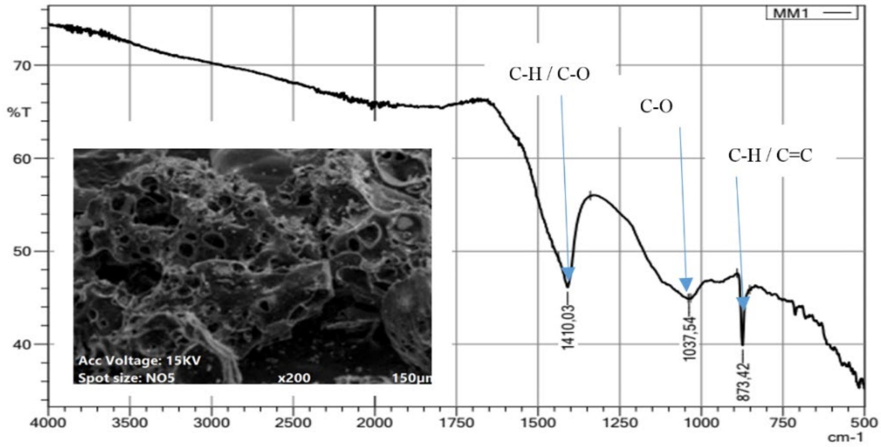

Figure 1. FTIR spectrum and SEM image of the algae activated carbon (ACA) used.

The particle size distribution of algae-derived activated carbon was analyzed (Figure 2). A predominance of particles in the 30–50 µm range was observed, consistent with literature values for powdered activated carbon from algae. The cumulative curve indicates that over 85% of particles are below 100 µm, which makes the material highly dispersible within polymer matrices and effective for adsorption processes. The chemical composition of algae-derived activated carbon is shown in Table 2.

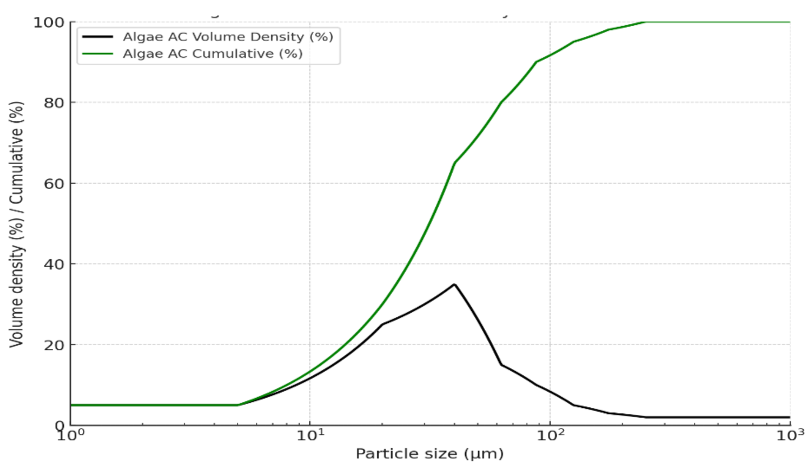

Figure 2. Particle size distributions of algae biochar (differential and cumulative curves).

Table 1. Algae-based biochar content elements (* includes mineral residues such as K, Ca, and Mg).

N°	Element	Content (wt.%)
1	Carbon (C)	59.83
2	Hydrogen (H)	7.27
3	Oxygen (O)	20.19
4	Nitrogen (N)	10.6 6
5	Others*	2.05

This composition reflects the protein- and mineral-rich nature of algae, which enhances surface reactivity and provides stability to the activated carbon.

The TGA curves indicate that algae-derived activated carbon is thermally more stable than raw algae (Figure 3).

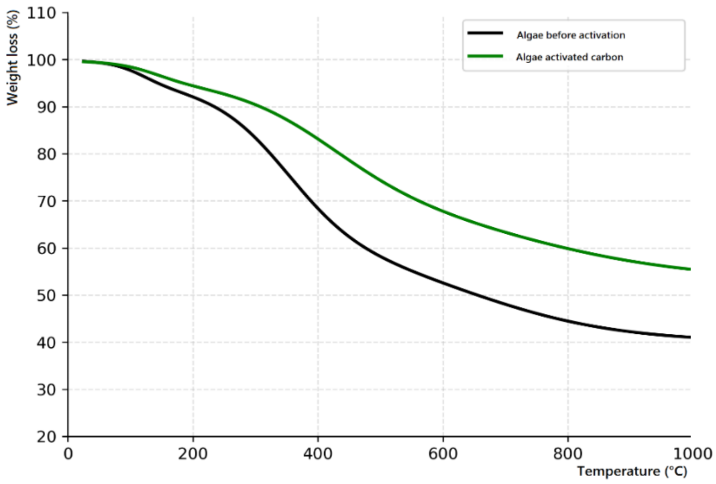

Figure 3. Thermogravimetric analysis curves of raw algae and algae-derived activated carbon.

Both materials show a small mass loss below ~150 °C due to moisture evaporation. Raw algae undergoes significant degradation between ~150 and 400 °C, associated with the decomposition of biopolymeric components such as polysaccharides and proteins, whereas the activated carbon exhibits a much slower mass loss, reflecting the removal of thermally labile species during activation. At higher temperatures, the activated carbon retains a higher residual mass, confirming the formation of a more stable carbonaceous structure.

XPS elemental composition of PU based composites.

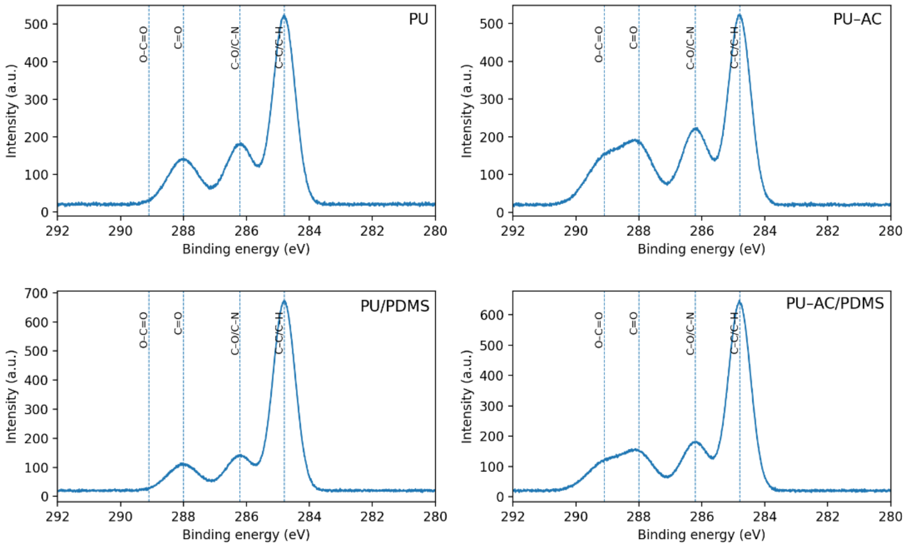

Figure 4. XPS C 1s Spectra: High-resolution C 1s spectra of all samples were deconvoluted into several components. The dominant peak at ~284.8 eV corresponds to C–C/C–H bonds of the polymer backbone and carbonaceous structures. Additional components at higher binding energies are assigned to C–O/C–N (~286.2 eV) and C=O (~287.8–288.2 eV) species associated with urethane linkages. In biochar-containing samples, an increased contribution at ~289.0–289.3 eV is observed, corresponding to O–C=O groups, which are characteristic of oxygenated functionalities introduced by chemical activation of biochar. These changes indicate an enrichment of polar surface groups without altering the fundamental polyurethane chemistry.

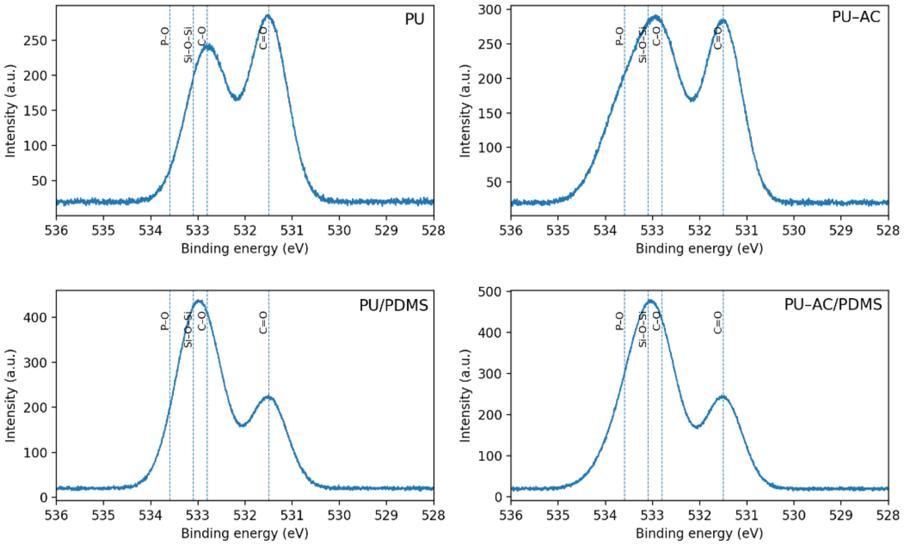

Figure 5. XPS O 1s spectra.

The O 1s spectra provide further insight into interfacial chemistry. Pristine PU shows two main components corresponding to carbonyl oxygen (C=O) and single-bonded oxygen (C–O) within the urethane structure. In PU–AC samples, an additional contribution attributed to P–O species appears, confirming the presence of phosphate-related functional groups on the activated biochar surface. PDMS-coated samples exhibit an extra O 1s component assigned to Si–O–Si bonds, characteristic of crosslinked siloxane networks. The coexistence of C–O, P–O, and Si–O species in PU–AC/PDMS demonstrates the successful combination of biochar incorporation and PDMS coating.

https://doi.org/10.3390/xxxxx

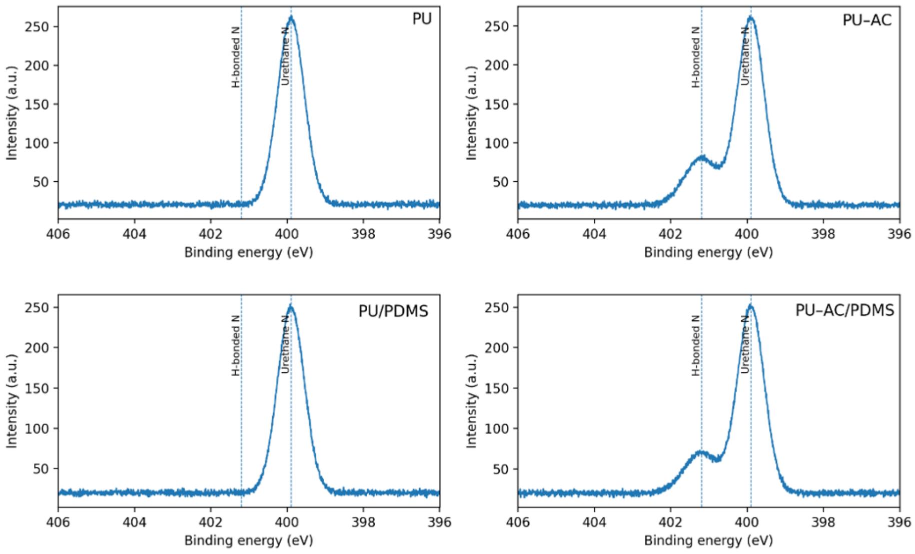

Figure 6. XPS N 1s spectra.

The N 1s spectra of all samples are dominated by a peak centered around ~399.8 eV, corresponding to urethane nitrogen (–NH–COO–). No additional nitrogen species or significant peak shifts are observed after biochar incorporation or PDMS coating. This similarity confirms that the polyurethane backbone remains chemically intact and that no new covalent bonds are formed involving nitrogen, supporting a non-reactive filler–matrix interaction mechanism.

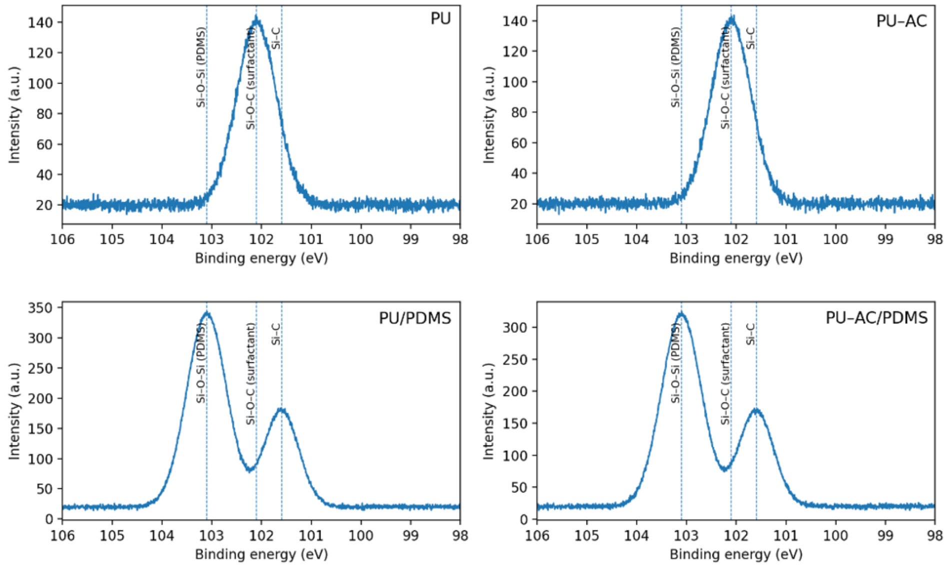

Figure 7. XPS Si 2p spectra.

High-resolution Si 2p spectra clearly distinguish between silicon originating from the silicone glycol surfactant and that from the PDMS coating. In pristine PU and PU–AC, the weak Si 2p signal is mainly associated with Si–O–C environments typical of silicone polyether surfactants. In contrast, PU/PDMS and PU–AC/PDMS exhibit a significantly stronger Si signal dominated by the Si–O–Si component at higher binding energy, characteristic of a crosslinked PDMS network. This confirms that PDMS forms a continuous surface layer rather than merely amplifying the pre-existing silicone signal from the surfactant.
